# Psychosocial Factors Influencing Breastmilk Production in Mothers After Preterm Birth: The Role of Social Support in Early Lactation Success—A Cross-Sectional Study

**DOI:** 10.3390/nu16223883

**Published:** 2024-11-14

**Authors:** Aleksandra Krawczyk, Agnieszka Czerwińska-Osipiak, Anna Weronika Szablewska, Wiktoria Rozmarynowska

**Affiliations:** Department of Obstetric and Gynaecological Nursing, Institute of Nursing and Midwifery, Medical University of Gdansk, Sklodowskiej-Curie 3a, 80-210 Gdansk, Poland; alekra@gumed.edu.pl (A.K.); aczerwinska@gumed.edu.pl (A.C.-O.); wiktoria.rozmarynowska@gumed.edu.pl (W.R.)

**Keywords:** breastfeeding, preterm infants, lactation, support

## Abstract

Background: Preterm birth is a major global health issue, associated with increased neonatal morbidity and mortality. Mothers of preterm infants face unique challenges, particularly with regard to lactation, due to the complexities of preterm neonatal care. Social support has been recognized as a factor in promoting successful breastfeeding, especially in vulnerable groups such as mothers of preterm infants. Objective: This study aimed to explore the psychosocial factors influencing breastfeeding in mothers following preterm birth, with a particular focus on the role of social support in early lactation success. Methods: A cross-sectional observational study was conducted between December 2022 and March 2023 at a tertiary referral hospital in Poland. The study included 130 women (71 mothers of preterm infants and 59 mothers of full-term infants) in the early postpartum period. Data were collected through a self-administered questionnaire and the Polish version of the Berlin Social Support Scales (BSSS). Lactation success was assessed based on the mother’s ability to initiate and maintain breastfeeding or expressed milk production. Statistical analyses included Mann–Whitney U tests, Pearson’s Chi-Square, and logistic regression to determine the association between social support and lactation outcomes. Results: The study revealed that mothers of preterm infants exhibited a greater need for social support compared with those who delivered at term. Social support, particularly perceived emotional and practical support, appeared to be significantly associated with successful early lactation. Women who received adequate social support were more likely to initiate breastfeeding early and maintain lactation. Conclusions: Psychosocial factors, particularly social support, play a critical role in the success of lactation among mothers of preterm infants. These findings underscore the need for comprehensive support systems for mothers of preterm infants to promote breastfeeding and improve neonatal health outcomes.

## 1. Introduction

Preterm birth, defined by the World Health Organization (WHO) as birth occurring before the completion of 37 weeks of gestation, remains a significant challenge in modern obstetrics. Globally, preterm births account for approximately 11% of all deliveries, with an estimated 15 million preterm infants born annually [1,2,3]. Preterm birth is responsible for 35% of neonatal deaths within the first 28 days of life. In Poland, demographic data from 2021 indicate that around 7% of births were preterm, including 1100 live births from pregnancies lasting less than 28 weeks. Despite extensive research, the exact causes of preterm birth remain elusive, though its impact on neonatal development is profound [4,5].

According to the 2015 CNoL (pol. Centrum Nauki o Laktacji, ang. Centre for the Study of Lactation) Report, in Poland, 98% of newborns are breastfed after birth. Unfortunately, this rate significantly decreases with each passing week. By the 6th month of life, only 42% of babies are still being breastfed. For premature newborns, 60% are breastfed at the time of discharge from level I and II reference hospitals. Level III reference hospitals show the lowest rates, with only 30% of preterm infants being breastfed at discharge [6,7].

Mothers of preterm infants represent a unique and vulnerable group. Not only do they experience the emotional and physiological changes associated with the postpartum period, but they also face the challenge of separation from their newborns. Preterm birth not only involves neonatal immaturity but also the unpreparedness of parents, particularly mothers, for early parenthood. Although the postpartum period for mothers of preterm infants follows a physiological pattern similar to that of those delivering at term, these women are at higher risk for emotional and psychological difficulties, with preterm birth often regarded as a traumatic event [8,9].

Lactation is a key physiological component of the postpartum period, particularly important for preterm infants due to the unique composition of breastmilk, which is tailored to the needs of the infant [10]. The hypothalamic–pituitary axis plays a crucial role in the initiation and maintenance of lactation. However, the process of lactation after preterm birth can be influenced by a multitude of factors, including neonatal health, maternal condition, and psychosocial influences. Early stimulation of lactation is critical, as delays in milk production are common after preterm deliveries, increasing the risk of lactation failure [11].

The psychosocial environment surrounding the mother is particularly important in determining lactation success. Social support, in particular, plays a pivotal role in both the initiation and continuation of breastfeeding. Mothers of preterm infants often require additional emotional and practical support due to the complexities associated with caring for a premature baby [12,13]. Yet, the relationship between psychosocial factors, including social support, and breastfeeding outcomes in preterm birth remains underexplored. Understanding these factors is crucial, as the benefits of breastfeeding for both mother and infant are well established, particularly in the context of preterm birth where breastmilk can significantly improve neonatal outcomes.

The aim of this study is to explore the psychosocial factors influencing breastmilk production in mothers after preterm birth, with a particular focus on the role of social support in the early success of lactation. This cross-sectional observational study seeks to fill a gap in the current literature by examining how perceived and received social support impacts breastfeeding outcomes. The results may have significant implications for clinical practice, providing insights that could inform the development of targeted interventions aimed at improving lactation support for mothers of preterm infants. By identifying key determinants of lactation success in this population, we hope to contribute to improved health outcomes for both mothers and their infants.

## 2. Materials and Methods

### 2.1. Study Design

The present study was a single-center, cross-sectional study among a group of 130 Polish women (in Poland, ethnic minorities constitute a relatively small portion of the overall population, so we did not differentiate between ethnic groups) in the early postpartum period. We followed the STROBE guidelines for cross-sectional studies [14]. All procedures were performed in accordance with the principles outlined in the Declaration of Helsinki of the World Medical Association (WMA) and approved by the Bioethics Commission of the Gdansk Medical University, no. NKBBN/730/2022 for studies involving humans.

### 2.2. Setting

The study was conducted in one tertiary referral hospital in the northern region of Poland (the classification system in Poland defines different levels of maternal care: level I focuses on managing physiologically normal pregnancies, childbirth, the postpartum period, and care for healthy newborns (with possible short-term pregnancy complications), level II provides for pregnancies with moderate complications, and level III is designated for the most complex and high-risk pregnancy cases). The period of data collection and patient eligibility was from December 2022 to March 2023.

### 2.3. Participants

All participants were informed about the study’s objectives and gave their voluntary consent to participate by indicating their agreement within the questionnaire. The principal investigator had a personal conversation with each mother of the children, explaining all concerns about the conduct of the study and the recruitment process. Mothers were invited to participate in the study while they were still in the maternity ward following childbirth. Mothers had the right to ask questions.

The study included 130 mothers (59 women after full-term births and 71 women after preterm births), hospitalized in the maternity wards of the study hospital, who met the inclusion criteria. 

A total of 157 surveys were distributed to postpartum women who met the inclusion criteria. Of these, 146 surveys were returned, and after excluding 16 surveys due to incomplete responses, 130 correctly completed questionnaires were included in the final analysis. Participants were recruited from the maternity and neonatal care wards, with the sample consisting of mothers who had delivered both preterm and full-term infants. The study focused on preterm births, but full-term mothers were included for comparison purposes.

The sample size calculation was based on statistical data regarding preterm births in Poland, as reported by the Central Statistical Office (GUS). In 2022, approximately 305,000 births were recorded, with around 7% of these being preterm. In order to determine the necessary sample size for the study, three groups were considered: postpartum women (*n* = 130), mothers of babies born at term (*n* = 59) and mothers of premature babies (*n* = 71). A significance level of α = 0.05 and a statistical power of 1 − β = 0.8 were assumed. To compare the two groups, a formula was used to calculate the sample size for the *t*-test. Assuming a common standard deviation of 10 and an expected difference between means of 5, the calculated minimum sample size was 63 participants in each group. The established number of participants provided adequate power for the test to detect significant differences between the groups analyzed.

**Figure 1:**
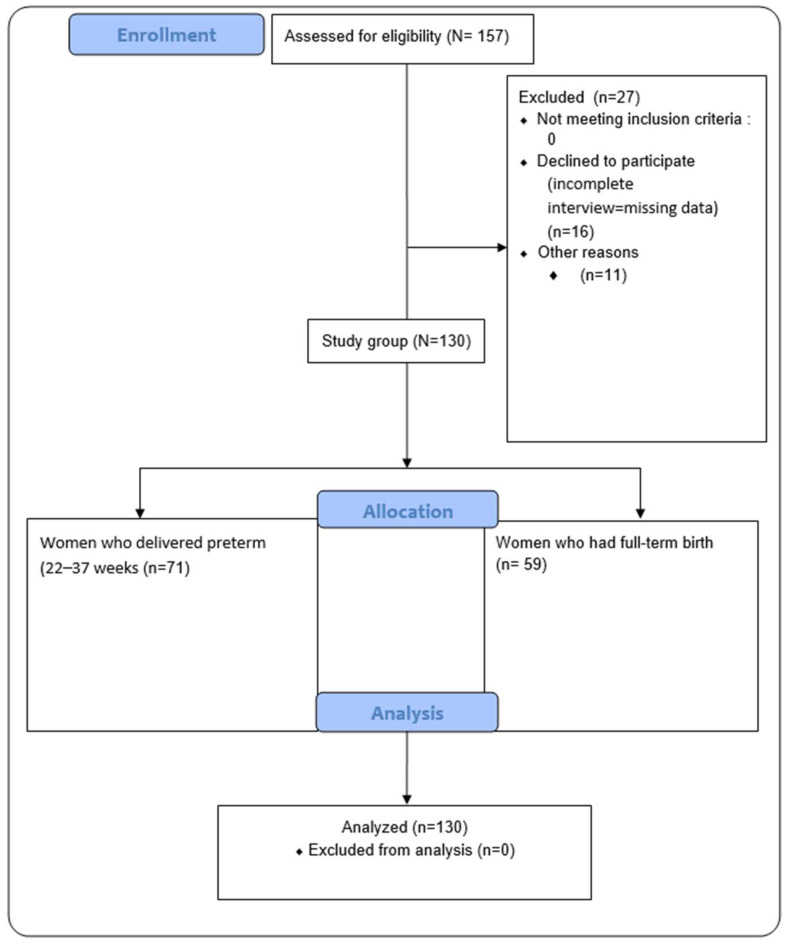
The flow of participants through the study.

The participants’ flow through the study is presented in Figure 1.

### 2.4. Inclusion and Exclusion Criteria

The inclusion criteria were women in early postpartum (up to 1 week after delivery), aged 18 years or older, who delivered either preterm or at term. Women who did not speak Polish were excluded from the study.

### 2.5. Data Collection Tools

Data were collected using a diagnostic survey method. The primary data collection tool was a self-administered questionnaire consisting of two parts:

Demographic and obstetric information: the first section of the questionnaire included 18 items that gathered demographic data (age, education level, etc.) and obstetric information, such as birth history and lactation details. This section was developed specifically for this study.

Berlin Social Support Scales (BSSS): The second part utilized a standardized tool, the Polish version of the BSSS, developed by Łuszczyńska et al. [15]. This instrument assesses various aspects of social support, including perceived available support, need for support, seeking support, received support, and protective-buffering support. The BSSS consists of 38 statements rated on a 4-point Likert scale (1 = completely untrue, 4 = completely true), with higher scores indicating greater levels of social support.

### 2.6. Variables and Outcome Measures

The primary outcome of interest was the success of early lactation, measured in terms of the mother’s ability to initiate and maintain breastfeeding or expressed milk production within the first week postpartum. Key independent variables included the following:

- Perceived social support: the extent to which mothers felt supported by family, friends, and healthcare professionals.
- Sociodemographic factors: age, education, and socioeconomic status.
- Obstetric and neonatal factors: mode of delivery, gestational age at birth, and neonatal health status.

### 2.7. Statistical Analysis

Data were analyzed using Statistica v12 (Advanced package with Plus set, PQStat Software v1.8., 2020) and Microsoft Excel 2007. The following statistical tests were applied:

Mann–Whitney U Test: A non-parametric test was used to compare the median levels of social support and lactation success between the two independent groups (preterm and full-term mothers).

Pearson’s Chi-Square Test: This test was used to examine the association between categorical variables, such as the relationship between social support levels and lactation success.

A *p*-value of <0.05 was considered statistically significant. Additionally, descriptive statistics, including means, medians, and standard deviations, were used to summarize the data.

Bivariate analyses (correlations) and multivariate analyses (logistic regression) were also used.

To examine the strength of the association between the different types of support in the study group (*n* = 130), J. Guilford’s classification was used. In the correlation analysis, all results were positive, meaning that an increase in one variable was associated with an increase in the other variable. The strength of the correlation is presented in the Results section, and the values were interpreted according to Guilford’s guidelines.

A logistic regression analysis was conducted to investigate the influence of sociodemographic variables and types of support on breastfeeding behavior. Logistic regression was chosen because the primary outcome variable—whether the mother is breastfeeding or expressing milk—is binary. This model allows for the estimation of the odds of lactation success based on a set of independent variables, including sociodemographic factors (e.g., age, education, infant birth weight) and various aspects of social support (e.g., perceived available support, currently received support, buffering support). The optimal regression model included 12 independent variables and a free expression. The model showed a moderately high quality of fit, obtaining fit index values of R^2^Pseudo = 0.39, R^2^Nagelkerke = 0.48, and R^2^Cox-Snell = 0.25. The model was statistically significant (*p* = 0.00016 in the likelihood ratio test), indicating a significant influence of the independent variables. In addition, the result of the Hosmer–Lemeshow test (*p* = 0.051) suggests no significant differences between the observed counts and the predicted probabilities, meaning that the model represents the empirical data well.

## 3. Results

### Characteristics of Study Group

A total of 130 postpartum women participated in the study, including 59 women with full-term deliveries and 71 women with preterm deliveries in the early postpartum period. Of these, 9% were births before 28 weeks of pregnancy, 12% between 28 and 31 weeks of pregnancy, and 34% between 32 and 36 weeks of pregnancy. Among preterm newborns, the largest group (49.3%, *n* = 35) had a birth weight of 1501–2500 g. Those with a birth weight of less than 1000 g accounted for 16.9%, those between 1001–1500 g comprised 1.4%, and those above 2500 g represented 32.4%. Notably, all full-term newborns had birth weights above 2500 g. The demographic and social data of the women participating in the study are presented in Table 1. It details information on age, education, place of residence, marital status and deliveries to date.

The study sought to analyze the different types of support among postpartum women and their impact on lactation using selected items of the Berlin Social Support Scales. A summary of the scores for both groups is presented in Table 2. It was shown that the women surveyed rated the perceived availability of support highly, which means that they had a subjective belief that they would be able to count on support from others in a crisis situation. The support currently received, i.e., that which the midwives were using at the time of completing the questionnaire, was also rated highly. The support currently received consists of forms of informational support, emotional support, instrumental support and overall satisfaction with the support.

Seeking support and the need for support, i.e., the existence of a need to use the support received in a difficult and crisis situation, were rated slightly lower in the respondents’ opinions. Buffer-protective support was rated low, meaning that respondents did not show a high need to hide negative information from relatives.

In the study group, the majority of women were positive about breastfeeding before delivery, as shown in Table 3. However, mothers after preterm births showed a greater lack of confidence. More than 14% (*n* = 10) of the respondents in this group answered that, despite their positive attitude, they did not believe in their abilities regarding breastfeeding.

In the study, the method of delivery differed between the preterm and term groups, with notable percentages for each. Among mothers who delivered preterm, 66% (*n* = 47) had vaginal deliveries, while approximately 34% had cesarean sections. In the term birth group, 56% (*n* = 33) had vaginal deliveries, and around 33.5% underwent cesarean sections. An analysis was conducted to examine whether lactation initiation among study participants was associated with the method of delivery. This relationship was assessed through Pearson’s Chi-Square tests, with results summarized in Table 4. The analysis revealed statistically significant findings (*p* < 0.05) in the group of preterm deliveries.

The findings demonstrate that assisted deliveries, including those using vacuum extraction or forceps, were associated with a lower frequency of lactation initiation within the first 24 h postpartum. Specifically, 54.9% (*n* = 39) of preterm mothers who had vaginal deliveries initiated lactation stimulation within the first day following birth. In contrast, mothers who underwent cesarean sections tended to start lactation stimulation using a breast pump sooner than those with vaginal deliveries.

Additional Pearson’s Chi-Square analyses were performed to explore the association between ongoing breastfeeding practices and the method of delivery. As indicated in Table 5, these analyses did not yield statistically significant results (*p* > 0.05), suggesting no clear relationship between current breastfeeding or expressed milk provision and delivery method in either the preterm or term groups.

To calculate the correlations in the study group (*n* = 130) between the different types of support, J. Guilford’s classification was used. The strength of the relationship between the variables is shown in Table 6, in which all correlations are positive—if the value of one variable increases, the value of the other variable also increases.

It has been shown that:

- If the perceived available support increases, the search for support also increases—weak correlation
- If the need for support increases, the search for support also increases—very high correlation
- If the support currently received increases, the perceived support available also increases—average correlation
- If the search for support increases, the support currently received also increases—weak correlation
- If the demand for support increases then buffer support also increases—weak correlation
- If support-seeking increases, then buffer-protective support also increases—weak correlation

Based on the model comparison analysis, from a statistical point of view, the optimal model to investigate the influence of socio-demographic data and support on breastfeeding or maternal pumping of breast milk for the newborn is the model with 12 independent variables and a free expression. Table 7 presents the logistic regression model in the study group (*n* = 130) for the variables analyzed.

**Figure 2:**
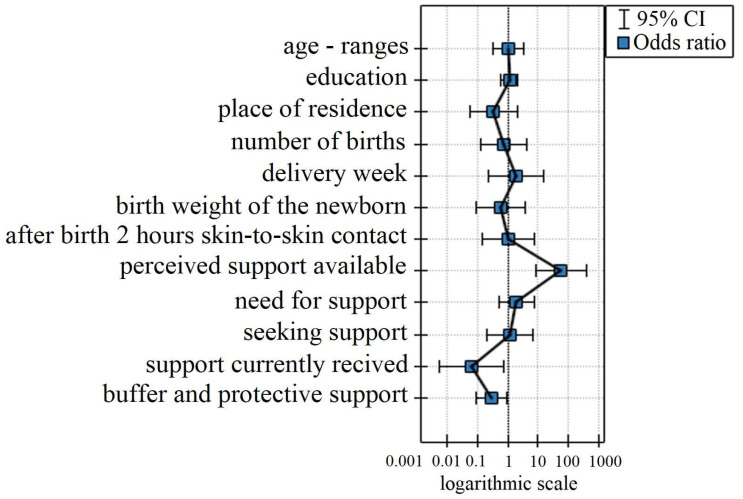
Variables as described by the odds ratio.

The chance that a mother will breastfeed or pump breastmilk for her newborn depends on the listed variables as described by the odds ratio in Figure 2.

It is shown that

- The higher the perception of available support, the greater the chance is that the mother will breastfeed or express breastmilk for the newborn [variable perception of available support: OR [95%CI] = 57.6 [8.22;404.31]]. This means that the more mothers who felt subjectively that they had access to support from those around them, the more likely they were to breastfeed or express milk.
- The greater the support currently received, the less likely the mother is to breastfeed or express breastmilk for the newborn [variable currently received support: OR [95%CI] = 0.06 [0.01;0.72]]. It is likely that women who had to use lactation support had problems that needed to be addressed, which equated to a lower chance of breastfeeding. The more frequent the problem requiring resolution and support, the lower the chance of breastfeeding.
- The greater the buffer-protective support, the less likely the mother was to breastfeed or express breast milk for the newborn [buffer-protective support variable: OR [95%CI] = 0.29 [0.09;0.95]]. This means that the more mothers wanted to hide negative news from their family, the less likely they were to breastfeed or express milk.

In the case of statistically insignificant variables, the confidence interval for the odds quotient contains a one, which means that these variables neither increase nor decrease the chance of the defect under study. Therefore, the resulting quotient cannot be interpreted in a way similar to that for statistically significant variables.

In order to verify whether women with preterm births present a higher rate of the need for social support and of seeking social support compared with women giving birth full term, a comparative analysis was performed using the Mann–Whitney U test. The results of these analyses showed that the time of delivery was statistically significantly associated with need for support (*p* = 0.000), support seeking (*p* = 0.000) and intensity of buffer-protective support (*p* < 0.01). It was confirmed that women with preterm births present a higher rate of needing and seeking social support compared to women giving birth at term. This means that postpartum women with preterm births show a higher need for support in a crisis situation, which is certainly the birth of a preterm baby. Mothers of newborns born prematurely seek support with greater frequency and to a greater extent.

The aim of the study was also to see if there was a relationship between social support and current breastfeeding. For this purpose, a comparative analysis with Mann–Whitney U tests was also performed. The results of these analyses were mostly found to be statistically insignificant (*p* > 0.05). The only statistically significant association of current breastfeeding or pumping found was with the perceived availability of support (*p* = 0.000).

It was confirmed that the higher the score in the domain of perceived available support, the more often women with preterm births breastfed and pumped breastmilk for their newborn. The more mothers of preterm infants had a perception of available support from others, the more often they breastfed/pumped.

In order to test whether attitudes towards breastfeeding were associated with a higher frequency of breastmilk feeding after preterm birth, analyses were performed using Pearson’s χ2 tests, and the results are shown in Table 8. The results of these analyses were found to be statistically significant (*p* < 0.05) in both groups.

It was confirmed that a positive attitude towards breastfeeding before delivery increased the frequency of breastfeeding or pumping breast milk in the group of women after both full-term and preterm deliveries. However, mothers of preterm births were more likely to lack confidence in their ability than mothers after a full-term birth.

## 4. Discussion

In light of the rising rates of preterm births worldwide, despite advances in perinatology, this issue requires further analysis. Statistics show that each year, approximately one million children die globally due to prematurity, representing a significant public health challenge [16]. There remains a high incidence of health complications among preterm infants, even with improvements in the survival rates of very low birth weight (VLBW) newborns [17].

Support for lactation and breastfeeding is a crucial element in the care of preterm infants. Therefore, promoting breastfeeding should be regarded as a form of medical therapy that benefits both infants and women’s reproductive health [18]. In the author’s research, it was observed that many factors influencing lactation development are psychosocial in nature.

The results of the analysis conducted in the author’s study indicate that perceived available support and current received support were rated highly by mothers (3.81; SD = 0.42 and 3.67; SD = 0.42), while buffering-protective support received a lower rating (2.11; SD = 0.6). These observations may suggest that patients who shielded their loved ones from negative information might have had less success with lactation. This phenomenon, confirmed by Wigert’s studies, highlights the need for diverse forms of psychosocial support for parents of preterm infants [13].

Breastfeeding rates in neonatal intensive care units (NICUs) in Poland remain low. To improve the situation, an Early Lactation Stimulation Program has been developed for neonatology and obstetrics centers at level III reference hospitals. The goal of this program is to standardize the actions of staff across all hospital units in the country. The implementation and application of these recommendations can help mothers initiate and maintain lactation. The team necessary for the implementation of the project includes parents, medical staff from neonatology and obstetrics departments, speech therapists, psychologists, physiotherapists, and lactation consultants such as International Board Certified Lactation Consultants (IBCLCs), Certified Lactation Counselors (CDLs), and lactation educators [7]. The author’s research also demonstrated a higher demand for social support among postpartum women after preterm births compared with those who delivered at term. This suggests that this group of women with preterm births should be prioritized in postpartum care [19].

Research by Mercan and Selcuk shows a connection between self-efficacy, levels of social support, and lactation outcomes [20]. In the analyzed study, it was proven that a higher level of perceived available support increases the likelihood of breastfeeding or pumping milk (*p* = 0.000). The actions of medical personnel should focus on women in this particular situation by offering care from midwives and psychologists and enabling contact with loved ones.

The separation of newborns from mothers, although still common, is a practice that can lead to toxic stress [21]. Research by Shengana et al. demonstrated that appropriately conducted “skin-to-skin” contact can help reduce anxiety and stress in preterm infants [22]. The author’s research found that 80% of mothers of preterm infants were separated from their child immediately after birth, underscoring the need for a change in the approach to newborn care.

Lactation is also influenced by the method of delivery. In Poland, 47% of deliveries are by caesarean section, making the country one of the highest in terms of operative delivery rates in Europe [23]. As noted in previous research, mothers who undergo C-sections often experience delays in lactogenesis due to postoperative recovery and reduced early contact with the newborn, which can contribute to lactation challenges [24]. Post-operative recovery for mothers who undergo caesarean section is often complicated by pain, limited mobility, and the need to remain in a supine position during the first 24 h, factors that can hinder the onset of lactation [25]. In this study, a significant portion of mothers who delivered preterm were likely to have undergone C-sections, as is common in high-risk pregnancies that often necessitate surgical delivery for neonatal health reasons. This finding aligns with the notion that the preterm cohort might show higher C-section rates, which in turn may impact breastfeeding initiation and maintenance, particularly without sufficient lactation support during the critical early postpartum period. As recruitment in the study spanned the first postpartum week, the variability in lactation initiation and duration was also likely influenced by the specific postpartum days of recruitment and evaluation. Research indicates that lactation initiation timing (particularly within the first 6 h post-birth) is a key factor for sustained breastfeeding success [26]. This study accounts for early postpartum factors, including the need for timely social and professional lactation support, which may mitigate some of the challenges associated with delayed lactation in C-section deliveries and preterm births. Further analysis of lactation success by postpartum day could add depth to understanding how timing and C-section rates specifically impact early lactation outcomes in preterm births.

In considering the challenges faced by mothers of preterm infants, it is essential to acknowledge their heightened demand for social and educational support. Craig et al. [27] emphasize the role of the family in the healing process for preterm infants, a finding supported by research by Bień et al. regarding the influence of partners on breastfeeding [28]. Similarly, the Academy of Breastfeeding Medicine recommends that education should encompass not only mothers but also the entire family, which is crucial in preventing early cessation of breastfeeding [29].

Given that mothers of preterm infants often experience feelings of guilt, as demonstrated by Thivierge’s studies, it is vital to support them in their lactation efforts and in the process of adapting to their new reality [30]. It is critical for medical personnel to be well-trained in communicating with parents and providing appropriate emotional support [31,32].

In discussing the findings, it is essential to consider the role of social pressure alongside social support in influencing lactation outcomes among mothers of preterm infants. Although our study focused on the positive aspects of social support, such as emotional and practical assistance, we recognize that certain forms of support might carry implicit or explicit pressure to initiate or sustain breastfeeding. Social pressure, particularly when aligned with societal norms and expectations regarding breastfeeding, may create an additional layer of psychosocial stress for mothers, especially those who face unique challenges due to preterm birth. Emerging literature suggests that social pressure can sometimes undermine maternal confidence or contribute to feelings of guilt and inadequacy, especially if mothers encounter difficulties with lactation or are unable to meet external expectations. This aspect is particularly pertinent in cases of preterm infants, where the medical and developmental needs may complicate breastfeeding efforts. Therefore, it is crucial for healthcare providers to balance support with sensitivity to avoid inadvertently exerting pressure on mothers, which may adversely impact their psychological well-being and lactation experience [33,34]. Future studies should aim to delineate the impact of social pressure as distinct from social support, examining how perceived pressure may interact with maternal mental health and lactation success. By further exploring these dynamics, healthcare interventions can be better tailored to address the complex social environment surrounding mothers of preterm infants, fostering supportive rather than pressuring approaches to breastfeeding promotion.

While the analysis of interactions between socio-economic variables and types of support could provide further valuable insights, we chose not to explore this aspect in the current study due to the complexity it would introduce and the focus on the primary research questions. However, we acknowledge that future studies may benefit from examining these interactions to better understand their influence on breastfeeding outcomes.

Another limitation of the current study is that it did not assess the impact of breastfeeding on maternal health, which could provide a more comprehensive view of the reciprocal relationships between breastfeeding and maternal well-being. Investigating these effects in future research would undoubtedly enrich our understanding of the broader implications of breastfeeding for mothers.

One of the strengths of this study is the relatively large sample size, particularly in the group of mothers of preterm infants, which allows for more reliable and generalizable findings regarding the psychosocial factors influencing lactation. Another key strength is that the research was conducted in a level III perinatal care center, where mothers of preterm infants are enrolled in an Early Lactation Stimulation Program, ensuring standardized lactation support. However, a potential limitation is the reliance on self-reported data, which may introduce recall bias or subjectivity, particularly regarding perceived support and lactation success.

In summary, lactation following preterm birth is a complex issue that necessitates specific support for mothers. The conclusions drawn from the conducted studies indicate a need for further research in this area to better understand and enhance the ways in which postpartum women can be supported, especially regarding factors influencing lactation success. Publishing the results of the author’s research could significantly expand the knowledge of interdisciplinary teams involved in the care of preterm infants and their families, highlighting the importance of a holistic approach to lactation support.

## 5. Conclusions

The findings from this research highlight that various factors—psychosocial, maternal, and neonatal—significantly influence lactation following preterm births. Mothers of preterm infants demonstrate a greater need for and actively seek social support compared with those delivering at term. Notably, the perception of available support correlates positively with the likelihood of breastfeeding or expressing milk. Prenatal education about the benefits of breastfeeding plays a crucial role in these decisions, while instrumental deliveries can hinder the initiation of lactation. Additionally, a positive attitude toward breastfeeding before delivery enhances postpartum breastfeeding rates. Conversely, mothers of preterm infants often exhibit lower confidence in their lactation abilities, and attempts to shield family members from negative information may diminish breastfeeding success. These findings underscore the importance of developing guidelines to promote breastfeeding among preterm infants through interdisciplinary collaboration, minimizing separations, and providing comprehensive education for families. Expanding research on lactation determinants is essential for public health and nutritional therapy for preterm infants, as it may uncover critical insights regarding the influence of separation on mothers’ milk production and overall lactation success.

## Figures and Tables

**Table 1:** Characteristics of the study group.

Class	Full-Term Birth		Preterm Birth	
Age	**N**	**%**	**N**	**%**
<25 years	14	23.73	14	19.72
25–30 years	21	35.59	34	47.89
>30 years	24	40.68	23	32.39
Education				
primary	3	5.08	4	5.63
middle school/lower	1	1.69	3	4.23
basic vocational	4	6.78	9	12.68
secondary	15	25.43	21	29.58
higher	36	61.02	34	47.88
Place of residence				
village	18	30.51	21	29.58
city	41	69.49	50	70.42
Marital status				
miss	17	28.81	22	30.99
married	41	69.50	47	66.19
widow	0	0	1	1.41
divorced	0	0	1	1.41
separated	1	1.69	0	0
Number of births				
one birth	30	50.85	32	45.07
two or more births	29	49.15	39	54.93

**Table 2:** Social support—summary.

		Full-Term Delivery	Preterm Birth
Perceived support available	Average	3.74	3.81
	SD	0.47	0.42
Demand for support	Average	2.48	3.59
	SD	0.85	0.52
Seeking support	Average	2.74	3.52
	SD	0.59	0.49
Current support received	Average	3.67	3.67
	SD	0.40	0.42
Buffer and protective support	Average	1.88	2.11
	SD	0.63	0.60

**Table 3:** Attitudes towards breastfeeding.

Full-Term Birth			Preterm Birth		
What Was Your Attitude to Breastfeeding Before Giving Birth?					
I wanted to	I wanted to	I didn’t want	I wanted to	I wanted to	I didn’t want
breastfeed		to breastfeed	breastfeed	breastfeed	to breastfeed
	breastfeed, but			but	
	I did not			I did not	
	believe in			believe in	
	my			my	
	abilities			abilities	
83.00%	11.90%	5.10%	78.90%	14.10%	7.00%

**Table 4:** Current breast milk feeding versus mode of birth.

	Delivery on Time				Preterm Birth			
Mode of Birth	Starting to Stimulate Lactation with a Breast Pump							
	up to 24 h after delivery	more than 24 h after delivery	no, because I didn’t want/need to	no, because no one has offered it to me	up to 24 h after delivery	more than 24 h after delivery	no, because I didn’t want/need to	no, because no one has offered it to me
Vaginal birth	30.30%	21.21%	27.27%	21.21%	53.19%	27.66%	12.77%	6.38%
Vacuum/forceps delivery	33.33%	50.00%	16.67%	0.00%	0.00%	0.00%	0.00%	100.00%
C-section	45.00%	35.00%	10.00%	10.00%	60.87%	26.09%	8.70%	4.35%
	35.59%	28.81%	20.34%	15.25%	54.93%	26.76%	11.27%	7.04%
	Pearson’s Chi^2: 6.54349, *df* = 6, *p* = 0.365132				Pearson’s Chi^2: 13.8865, *df* = 6, *p* = 0.030930			

**Table 5:** Current breast milk feeding versus mode of pregnancy completion.

	Delivery on Time		Preterm Birth	
Mode of Birth	Current Breastfeeding or Pumping for Newborn			
	YES	NO	YES	NO
Vaginal birth	84.85%	15.15%	91.49%	8.51%
Vacuum/forceps delivery	66.67%	33.33%	100.00%	0.00%
C-section	90.00%	10.00%	86.96%	13.04%
	84.75%	15.25%	90.14%	9.86%
	Pearson’s Chi^2: 1.94442, *df* = 2, *p* = 0.378247		Pearson’s Chi^2: 0.467969, *df* = 2, *p* = 0.791374	

**Table 6:** Correlations.

	Perceived Available Support	Demand for Support	Seeking Support	Support Currently Received	Buffer and Protective Support
Perceived available support	1	0.1343	0.2114	0.3102	0.0338
	*p*= ---	*p* = 0.128	*p* = 0.016	*p* = 0.000	*p* = 0.702
Demand for support	0.1343	1	0.8447	0.1389	0.2627
	*p* = 0.128	*p* = ---	*p* = 0.00	*p* = 0.115	*p* = 0.003
Seeking support	0.2114	0.8447	1	0.182	0.268
	*p* = 0.016	*p* = 0.00	*p* = ---	*p* = 0.038	*p* = 0.002
Support currently received	0.3102	0.1389	0.182	1	0.0631
	*p* = 0.000	*p* = 0.115	*p* = 0.038	*p* = ---	*p* = 0.476
Buffer and protective support	0.0338	0.2627	0.268	0.0631	1
	*p* = 0.702	*p* = 0.003	*p* = 0.002	*p* = 0.476	*p* = ---

**Table 7:** Model for the analyzed variables.

	b	error b	−95% CI	+95% CI	stat. Walda	*p*-Value	Odds Ratio	−95% CI	+95% CI
w. free	−0.37	5.83	−11.79	11.06	0.00	0.949835	0.69	0.00	63617.70
age—ranges	0.05	0.61	−1.14	1.24	0.01	0.934441	1.05	0.32	3.45
education	0.10	0.33	−0.55	0.74	0.09	0.76809	1.10	0.58	2.10
place of residence	−1.12	0.92	−2.93	0.69	1.47	0.225815	0.33	0.05	2.00
number of births	−0.31	0.87	−2.02	1.39	0.13	0.716731	0.73	0.13	4.00
week of childbirth	0.65	1.07	−1.44	2.74	0.37	0.542988	1.91	0.24	15.45
mass neonatal birth rate	−0.55	0.94	−2.39	1.30	0.34	0.560528	0.58	0.09	3.65
two-hour skin-to-skin contact	0.02	1.01	−1.95	2.00	0.00	0.982395	1.02	0.14	7.36
perceived support available	4.05	0.99	2.11	6.00	16.65	0.000045	57.65	8.22	404.31
demand for support	0.64	0.68	−0.70	1.98	0.87	0.350798	1.89	0.50	7.23
search for support	0.12	0.89	−1.62	1.86	0.02	0.891998	1.13	0.20	6.41
currently support received	−2.78	1.25	−5.23	−0.33	4.94	0.02629	0.06	0.01	0.72
support buffer-protective	−1.22	0.60	−2.39	−0.05	4.21	0.040252	0.29	0.09	0.95

**Table 8:** Feeding attitudes before childbirth versus current breastfeeding.

	Full-Term Birth			Preterm Birth		
Current Breastfeeding/ Pumping	Attitudes Towards Breastfeeding Before Childbirth					
	I wanted to breastfeed	I wanted to breastfeed, but I didn’t believe in my abilities	I didn’t want to breastfeed	I wanted to breastfeed	I wanted to breastfeed, but I didn’t believe in my abilities	I didn’t want to breastfeed
YES	96.00%	4.00%	0.00%	84.38%	9.38%	6.25%
NO	11.11%	55.56%	33.33%	28.57%	57.14%	14.29%
General	83.05%	11.86%	5.08%	78.87%	14.08%	7.04%
	Pearson’s Chi^2: 40.3715. *df* = 2. *p* = 0.000001			Pearson’s Chi^2: 13.2921. *df* = 2. *p* = 0.001300		

## Data Availability

The original contributions presented in the study are included in the article, further inquiries can be directed to the corresponding author.
